# Robustness of Massively Parallel Sequencing Platforms

**DOI:** 10.1371/journal.pone.0138259

**Published:** 2015-09-18

**Authors:** Pınar Kavak, Bayram Yüksel, Soner Aksu, M. Oguzhan Kulekci, Tunga Güngör, Faraz Hach, S. Cenk Şahinalp, Turkish Human Genome Project, Can Alkan, Mahmut Şamil Sağıroğlu

**Affiliations:** 1 Department of Computer Engineering, Boğaziçi University, İstanbul, Turkey; 2 Advanced Genomics and Bioinformatics Research Group (İGBAM), BİLGEM, The Scientific and Technological Research Council of Turkey (TÜBİTAK), Gebze, Kocaeli, Turkey; 3 TÜBİTAK - MAM - GMBE (The Scientific and Technological Research Council of Turkey, Genetic Engineering and Biotechnology Institute), Gebze, Kocaeli, Turkey; 4 School of Computing Science, Simon Fraser University, Burnaby, BC, Canada; 5 Department of Computer Engineering, Bilkent University, Ankara, Turkey; The University of Hong Kong, HONG KONG

## Abstract

The improvements in high throughput sequencing technologies (HTS) made clinical sequencing projects such as ClinSeq and Genomics England feasible. Although there are significant improvements in accuracy and reproducibility of HTS based analyses, the usability of these types of data for diagnostic and prognostic applications necessitates a near perfect data generation. To assess the usability of a widely used HTS platform for accurate and reproducible clinical applications in terms of robustness, we generated whole genome shotgun (WGS) sequence data from the genomes of two human individuals in two different genome sequencing centers. After analyzing the data to characterize SNPs and indels using the same tools (BWA, SAMtools, and GATK), we observed significant number of discrepancies in the call sets. As expected, the most of the disagreements between the call sets were found within genomic regions containing common repeats and segmental duplications, albeit only a small fraction of the discordant variants were within the exons and other functionally relevant regions such as promoters. We conclude that although HTS platforms are sufficiently powerful for providing data for first-pass clinical tests, the variant predictions still need to be confirmed using orthogonal methods before using in clinical applications.

## Introduction

The robustness and the reproducibility are the sine qua non of every data intended to be used for clinical applications. These factors have been the main issue hindering large scale applicability of array-based technologies for clinics. High throughput sequencing (HTS) offers alternative solutions to array based technologies with respect to genotyping, and HTS data are considered to be more robust and comprehensive. The performance of HTS platforms has been tested in various studies [1–3], but the robustness of HTS platforms still need to be systematically assessed. More specifically, it is of crucial importance to obtain accurate single nucleotide polymorphism (SNP), indel, and structural variation (SV) call sets in the sense that the calls made for specific SNPs or SVs should be solely dependent on the actual genotypes of sequenced individuals but not the location, time, or the platform of choice of the study.

Here we investigate the robustness of the Illumina HiSeq platform, currently the most widely used HTS technology in genome sequencing. In order to achieve this, we resequenced the genomes of two individuals from the Turkish Genome Project [4] twice. The two genomes were previously sequenced once [4], using the Illumina HiSeq 2000 platform in BGI Shenzhen, and a second time through the same platform set up at the Turkish Advanced Genomics and Bioinformatics Research Group (TÜBİTAK İGBAM). Although the same model sequencing machines were used, roughly the same level of coverage was achieved, and identical tools were used with identical parameters, independent analysis of the SNP and indel calls revealed significant number of differences between the two trials. In particular, we noticed that roughly 280 thousand of the 3 million SNPs genotyped by the GATK [5] tool in one trial (e.g. BGI) or the other (e.g. TÜBİTAK) are unique to only one callset—implying that the reproducibility rate of SNP calls is ∼ 92%. Interestingly, the multisample calling option of GATK that jointly analyzes two WGS datasets simultaneously does not seem to substantially improve the reproducibility and thus accuracy of the results. In this study, we explore the “sources” of this loss of accuracy as a function of both quality scores and coverage levels in each of the samples. Although increase in coverage levels in each sample typically decreases the differences between the GATK calls for specific loci on the two samples, there are still some cases in which differences can not be attributed to low coverage or quality score differences.

Our main contribution in this paper is a detailed investigation of the types and causes of exclusive variants within the call sets that are expected to be substantially the same. In addition, we try to identify strategies to handle such discrepancies when there is a second WGS dataset generated from the genome of the same donor. With further technological advancements and the cost improvements, sequencing a sample many times can be expected to be prevalent, as storing the data may become more expensive than resequencing the same sample. Here the same donor sample is sequenced twice, to evaluate the outcome of this highly possible situation in the future. For such cases, when there are more than one WGS sequence of the same donor, we state our remarks on how to exploit all the data fruitfully. In Section 1, we describe the methods used in the study. In Section 2, we present the results of the study and show the shared and exclusive sets of different SNP groups. And finally, in Section 3, we provide our remarks on the results and conclude.

## 1 Methods

### 1.1 DNA Samples and Ethics Statement

Genomic DNA from two individuals were collected and purified in 2011, only once from the blood of two volunteers for a previously published study [4]. The source (i.e. blood), DNA extraction time and location are the identical. As indicated in [4], institutional review board permission was obtained from INAREK (Committee on Ethical Conduct in Studies Involving Human Subjects at the Boğaziçi University) before data collection, and all participants including those that are included in this study provided informed consent.

### 1.2 Sequencing

The genomes of the two individuals were already sequenced using Illumina HiSeq2000 in 2011 at BGI Shenzhen [4]. The same samples were resequenced for a second time using another Illumina HiSeq2000 in 2012 at the TÜBİTAK İGBAM located in Kocaeli, Turkey. For the first sequencing data set, DNA samples were fragmented to 500bp, and paired-end sequencing data were generated with a read length of 90bp. For the second sequencing experiment at TÜBİTAK, we used the same protocols and sheared the DNA to 500bp fragments, and sequenced 104bp paired-end reads. In the remainder of the paper, we refer to the data generated at BGI as *S*_1*B*_ (first individual) and *S*_2*B*_ (second individual), and the data generated at TÜBİTAK for the same individuals as *S*_1*T*_ and *S*_2*T*_.

### 1.3 Alignment, coverage, GC content

To discover SNPs and short indels, we mapped the reads to the human reference genome (NCBI GRCh37) using the BWA aligner (version 0.6.2) [6], in paired-end mode (“sampe”) and default options. We converted the mapping output to sorted, duplicate-removed, and indexed BAM files using SAMtools [7]. We calculate the expected coverage as:

$$\begin{array}{c} \frac{\text{num}\_\text{mapped}\_\text{reads}\times \text{read}\_\text{length}}{2,897,310,462\;\text{(number}\;\text{of}\;\text{non-N}\;\text{bases}\;\text{in}\;\text{GRCh37)}} \end{array}$$

Next, SAMtools and BEDtools [8] were used to calculate the effective coverage:

$$\begin{array}{c} \frac{\left(\sum_{i=1}^{\text{num}\_\text{bases}}\text{Coverage}{}_{i}\right)}{\text{num}\_\text{bases}} \end{array}$$

Finally, we used the FASTQC tool (version 0.10.1) [9] to collect basic statistics of the genomic sequence data (Table 1).

**Table 1:** Summary of the sequence datasets.

Dataset	Number of reads	Read length	Expected Coverage	Number of mapped reads	Effective Coverage	GC%
*S*_1*T*_	1,401,819,290	104	45.6X	1,366,858,600	42.3X	42%
*S*_1*B*_	1,394,524,622	90	41.5X	1,272,512,132	37.6X	39%
*S*_2*T*_	934,050,130	104	31.3X	914,763,337	29.56X	43%
*S*_2*B*_	1,793,560,406	90	53.4X	1,688,991,592	49.2X	41%

Basic statistics of the two samples (*S*_1_, *S*_2_) sequenced at two different centers. *S*_1*T*_ refers to sample *S*_1_ sequenced at TÜBİTAK, where the dataset *S*_1*B*_ was generated from the same sample at BGI. Similarly, datasets from sample *S*_2_ are denoted as *S*_2*T*_ and *S*_2*B*_.

### 1.4 Variant calling

#### SNP and indel detection

After the initial alignment and the PCR-duplicate removal, we realigned the indel-containing reads to the reference genome using GATK Realigner tool. We then used the GATK UnifiedGenotyper tool to generate the SNP and indel call sets. We also used the GATK HaplotypeCaller as an alternative approach for variant calling. Next, we eliminated likely false positives using the GATK Variant Quality Score Recalibration (VQSR) tool with GATK resource bundle v2.5. Finally, we further filtered the call sets using the GATK VariantFiltration to remove low confidence calls (SnpCluster filter to remove SNPs if there are more than 3 SNPs in a 10 bp window). We applied the same variant calling pipeline to each of the four datasets separately: *S*_1*B*_, *S*_1*T*_, *S*_2*B*_ and *S*_2*T*_.

#### Pooled SNP and indel calling

As a second experiment, we tested whether pooling data from multiple sequencing runs for the same samples improve callset reproducibility. Our main question here was to understand if the slight differences in the coverage and depth of the datasets could be ameliorated by merging data for discovery, and if this would improve genotyping accuracy. For this purpose, we applied the SNP/indel detection pipeline to both samples by pooling two sequencing datasets (i.e. *S*_1*BT*_, and *S*_2*BT*_) generated at BGI and TÜBİTAK.

However, we named the two datasets from the same sample as if they were generated from different genomes. In the remainder of the paper, we denote the SNP/indels genotyped within the BGI data from *S*_1_ as *B*_1_, and the SNP/indels genotyped within the TÜBİTAK data from *S*_1_ as *T*_1_ for this experiment. Similarly, we have *B*_2_ and *T*_2_ for the sample *S*_2_.

### 1.5 Variant annotation

We used the ANNOVAR [10] tool (version 2013-02-21) to annotate SNPs and indels.

### 1.6 Data Availability

We had previously deposited the sequence reads obtained from BGI to the SRA read archive (SRP021510). Primary run IDs relevant to this study are: SRR839600 for *S*_1*B*_ and SRR849493 for *S*_2*B*_. Datasets generated at TÜBİTAK are also available as “secondary sequencing” data sets with sample IDs SRR2128004 and SRR2128088 respectively within the same SRA archive. We also released our scripts we used to map the reads and call the variants at https://github.com/pinarkavak/robust, and the VCF files for the call sets are available at http://alkanlab.org/paper-data/Kavak_RobustNGS/.

## 2 Results

### 2.1 Read length, coverage, GC content

We provide the basic analysis of the input data sets in Table 1. Briefly, we generated a total of more than 5.5 billion reads, equivalent to ∼ 530 Gbps, where the effective sequence coverage per data set ranged from 29.5*X* to 49.2*X*. The reads sequenced at TÜBİTAK (*S*_1*T*_ and *S*_2*T*_) were 14bp longer than the reads sequenced at BGI (*S*_1*B*_ and *S*_2*B*_), and the GC contents were similar (Table 1).

### 2.2 Call Sets and comparisons

#### SNP and indel discovery

We generated SNP call sets for each sample and for pooled data sets (Methods). 4 SNP call sets: *S*_1*T*_, *S*_1*B*_, *S*_2*T*_, *S*_2*B*_; and 2 pooled call sets for *S*_1_ and *S*_2_, denoted as *S*_1*BT*_, *S*_2*BT*_ were generated. 3 call sets per sample (i.e., *S*_1*T*_, *S*_1*B*_, and *S*_1*BT*_ for *S*_1_ and *S*_2*T*_, *S*_2*B*_, and *S*_2*BT*_ for *S*_2_) were compared with each other to quantify and characterize any differences. The SNP and indel statistics are summarized in Table 2. The SNP and indel statistics that were obtained by HaplotypeCaller are also shown in Table 3.

**Table 2:** SNPs and indels discovered using UnifiedGenotyper.

	SNPs		Indels	
	Total	Novel^1^	Total	Novel^1^
*S*_1*T*_	3,320,545	40,936	34,407	430
*S*_1*B*_	3,356,829	60,596	132,144	2,076
*S*_1*BT*_	3,340,498	55,408	80,950	1,227
*S*_2*T*_	3,277,433	46,448	56,189	756
*S*_2*B*_	3,346,221	55,753	54,229	529
*S*_2*BT*_	3,393,037	98,383	32,743	502

^1^Compared to dbSNP138

**Table 3:** SNPs and indels discovered using HaplotypeCaller.

	SNPs		Indels	
	Total	Novel^1^	Total	Novel^1^
*S*_1*T*_	3,540,735	57,905	614,241	35,624
*S*_1*B*_	3,504,854	58,578	668,779	41,558
*S*_1*BT*_	3,569,295	59,510	739,347	50,617
*S*_2*T*_	3,463,094	60,344	589,891	34,249
*S*_2*B*_	3,539,933	79,869	718,734	44,571
*S*_2*BT*_	3,613,663	72,099	217,365	57,056

^1^Compared to dbSNP138

***Separately generated call sets***. Briefly, after potential false positive removal (Methods), we observed approximately 95% agreement between the pairs of SNP call sets generated from both genomes (Table 4). The indel call sets showed a larger discrepancy, where only 18%-68% of each callset were shared with the other two call sets (Table 4). The number of shared and discrepant SNP and indels of HaplotypeCaller are shown in Table 5.

**Table 4:** Comparisons of total and novel SNP and indel call sets generated from the genomes of *S*_1_ and *S*_2_. *S*_1*B*_, *S*_1*T*_, *S*_1*BT*_: *S*_1_ calls from BGI, TÜBİTAK, and pooled datasets using UnifiedGenotyper; *S*_2*B*_, *S*_2*T*_, *S*_2*BT*_: *S*_2_ calls from BGI, TÜBİTAK, and pooled datasets, respectively.

	SNPs		Indels	
	Total	Novel	Total	Novel
*S*_1*B*_ ∩ *S*_1*T*_ ∩ *S*_1*BT*_	3,167,254	36,273	23,293	232
*S*_1*B*_ \ *S*_1*T*_ \ *S*_1*BT*_	75,839	16,073	67,478	1,239
*S*_1*T*_ \ *S*_1*B*_ \ *S*_1*BT*_	56,906	1,444	3,525	56
*S*_1*BT*_ \ *S*_1*B*_ \ *S*_1*T*_	22,737	8,896	11,647	300
(*S*_1*B*_ ∩ *S*_1*T*_) \ *S*_1*BT*_	29,807	615	1,476	26
(*S*_1*B*_ ∩ *S*_1*BT*_) \ *S*_1*T*_	83,929	7,635	39,897	579
(*S*_1*T*_ ∩ *S*_1*BT*_) \ *S*_1*B*_	66,578	2,604	6,113	116
*S*_2*B*_ ∩ *S*_2*T*_ ∩ *S*_2*BT*_	3,164,900	42,518	12,823	93
*S*_2*B*_ \ *S*_2*T*_ \ *S*_2*BT*_	40,492	4,899	22,599	258
*S*_2*T*_ \ *S*_2*B*_ \ *S*_2*BT*_	62,748	46,415	34,980	581
*S*_2*BT*_ \ *S*_2*B*_ \ *S*_2*T*_	62,029	2,314	3,567	219
(*S*_2*B*_ ∩ *S*_2*T*_) \ *S*_2*BT*_	12,972	251	5,420	35
(*S*_2*B*_ ∩ *S*_2*BT*_) \ *S*_2*T*_	127,857	8,085	13,387	143
(*S*_2*T*_ ∩ *S*_2*BT*_) \ *S*_2*B*_	37,532	1,365	2,966	47

**Table 5:** Comparisons of total and novel SNP and indel call sets generated from the genomes of *S*_1_ and *S*_2_. *S*_1*B*_, *S*_1*T*_, *S*_1*BT*_: *S*_1_ calls from BGI, TÜBİTAK, and pooled datasets using HaplotypeCaller; *S*_2*B*_, *S*_2*T*_, *S*_2*BT*_: *S*_2_ calls from BGI, TÜBİTAK, and pooled datasets, respectively.

	SNPs		Indels	
	Total	Novel	Total	Novel
*S*_1*B*_ ∩ *S*_1*T*_ ∩ *S*_1*BT*_	3,373,868	43,693	552,114	22,090
*S*_1*B*_ \ *S*_1*T*_ \ *S*_1*BT*_	36,182	7,005	7,863	6,189
*S*_1*T*_ \ *S*_1*B*_ \ *S*_1*BT*_	55,145	6,663	9,729	3,735
*S*_1*BT*_ \ *S*_1*B*_ \ *S*_1*T*_	25,347	2,418	27,621	5,919
(*S*_1*B*_ ∩ *S*_1*T*_) \ *S*_1*BT*_	18,223	1,015	794	235
(*S*_1*B*_ ∩ *S*_1*BT*_) \ *S*_1*T*_	76,581	6,865	108,008	13,044
(*S*_1*T*_ ∩ *S*_1*BT*_) \ *S*_1*B*_	93,499	6,534	51,604	9,564
*S*_2*B*_ ∩ *S*_2*T*_ ∩ *S*_2*BT*_	3,334,025	46,783	543,893	22,332
*S*_2*B*_ \ *S*_2*T*_ \ *S*_2*BT*_	35,153	18,073	4,807	1,762
*S*_2*T*_ \ *S*_2*B*_ \ *S*_2*BT*_	52,188	8,034	16,981	6,611
*S*_2*BT*_ \ *S*_2*B*_ \ *S*_2*T*_	43,596	10,903	54,639	9,291
(*S*_2*B*_ ∩ *S*_2*T*_) \ *S*_2*BT*_	5,797	600	687	175
(*S*_2*B*_ ∩ *S*_2*BT*_) \ *S*_2*T*_	164,958	14,413	169,347	20,302
(*S*_2*T*_ ∩ *S*_2*BT*_) \ *S*_2*B*_	71,084	4,927	28,330	5,131

**Fig 1:**
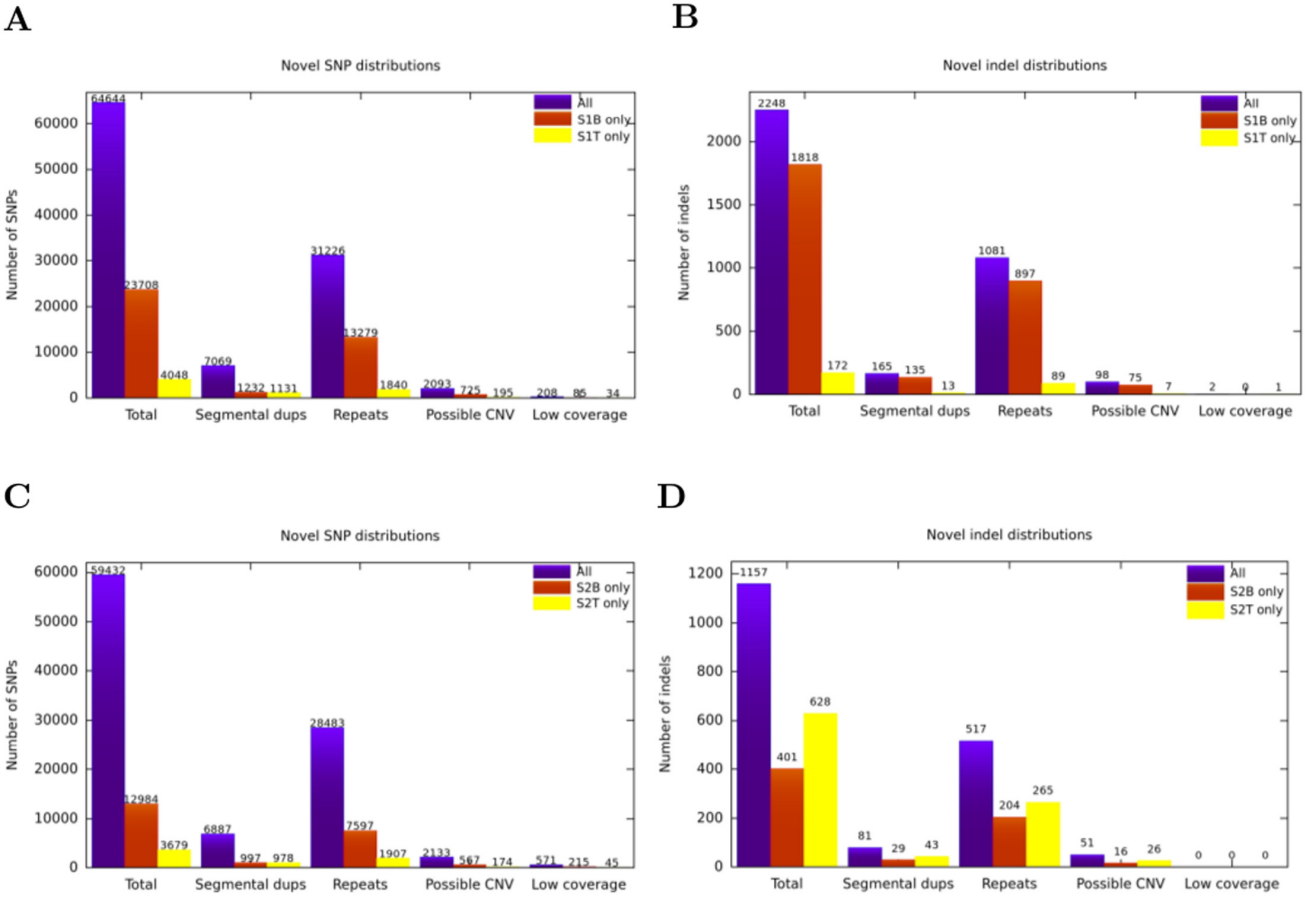
Underlying sequence content of novel SNP and indel calls. A) SNPs and B) indels in the genome of *S*_1_. C) SNPs and D) indels in the genome of *S*_2_.

To understand the causes of different calls from the same genomes, we investigated the underlying sequence content of the discrepancies of novel SNP and indel calls in detail. First, we downloaded the human reference genome annotations (segmental duplications and common repeats) from the UCSC genome browser (http://genome.ucsc.edu), CNV call sets from the 1000 Genomes Project [11]. We then calculated and annotated the number of novel SNPs and indels (Fig 1, Fig 1A and 1C, Fig 1B and 1D). We found that 46%-59% of discrepant novel SNP calls intersected with common repeats, and 5%-28% intersected with segmental duplications. In addition, a 3%-5% of the discrepant calls were found within the CNV regions reported in the 1000 Genomes Project [11], and 0.3%-0.8% were discovered in low coverage areas (< 5X). Analysis of the discrepant indel calls yielded similar results (Fig 1B and 1D). Next, we investigated the types of common repeats for the discrepancies. The majority of discrepant calls were found to be within Alu and L1 repeats (Tables 6 and 7). The incongruent calls within satellites and low complexity repeats were negligible. In addition, a close look to Alu and L1 subfamilies revealed that the number of discrepant calls peaked at ∼ 10% sequence divergence from consensus sequences, also showing negligible differences at recent and distant mobile element insertion loci (data not shown). Both of these observations can be explained by low mapping quality within these regions, causing the GATK VQSR algorithm to filter out such calls.

**Table 6:** Detailed view of novel SNP and indel distributions of *S*_1_ that map to common repeats.

	SNPs			Indels		
	All *S*_1_	*S*_1*B*_ only	*S*_1*T*_ only	All *S*_1_	*S*_1*B*_ only	*S*_1*T*_ only
Total	31,226	13,279	1,840	1,081	897	89
SINE/Alu	8,911	4,175	706	204	196	5
LINE/L1	8,779	3,581	332	415	330	33
LTR/ERV	5,370	2,022	263	84	74	4
Low compl.	429	196	55	63	41	11
Satellite	237	89	14	9	7	0
Simple rep.	1,605	1,011	312	151	118	27
Other	5,895	2,205	158	155	131	9

**Table 7:** Detailed view of novel SNP and indel distributions of *S*_2_ that map to common repeats.

	SNPs			Indels		
	All *S*_2_	*S*_2*B*_ only	*S*_2*T*_ only	All *S*_2_	*S*_2*B*_ only	*S*_2*T*_ only
Total	28,483	7,597	1,907	517	204	265
SINE/Alu	9,499	4,048	507	71	45	24
LINE/L1	7,396	1,331	511	208	71	112
LTR/ERV	4,360	434	221	66	20	38
Low compl.	653	399	59	32	17	12
Satellite	260	61	29	0	0	0
Simple rep.	1,489	784	410	54	26	27
Other	4,826	540	170	86	25	52

The significance of the discrepant SNP and indels in terms of predicted functionality was more closely investigated (Table 8). We found that 88%-95% of the discrepant SNP calls mapped to intergenic and intronic regions, where a 3.5%-4.5%- were predicted to be within coding exons, and ncRNAs. Indels showed similar properties, where only 0–3 of them were predicted to incur frameshifts.

**Table 8:** Distribution of discrepant novel SNP-indels of *S*_1_ and *S*_2_ over gene regions.

	Novel discrepant SNP-Indels of *S*_1_				Novel discrepant SNPs-Indels of *S*_2_			
	*S*_1*T*_		*S*_1*B*_		*S*_2*T*_		*S*_2_ *B*	
	SNP	Indel	SNP	Indel	SNP	Indel	SNP	Indel
Total	4,048	172	23,708	1,818	3,679	628	12,984	401
intergenic	2,191	107	13,451	1,029	2,261	358	6,470	249
intronic	1,506	50	8,899	694	1,196	233	5,016	126
upstream	62	2	139	10	34	2	467	4
downstream	44	1	144	8	28	2	89	3
UTR5	33	0	36	1	5	1	228	1
UTR3	29	3	199	17	21	5	96	5
exonic nonsyn	26	0	129	0	5	0	131	0
exonic syn	24	0	47	0	7	0	42	0
exonic stopgain	0	0	5	0	0	0	0	0
exonic unknown	0	0	1	0	0	0	4	0
exonic	0	0	1	0	0	0	0	0
ex. frmshift del^1^	0	0	0	1	0	1	0	0
ex. nonfrmshift del	0	0	0	1	0	0	0	0
ex. nonfrmshift ins	0	0	0	1	0	0	0	0
splicing	1	0	13	1	2	0	31	0
ncRNA intronic	114	9	609	55	116	26	357	12
ncRNA exonic	17	0	33	0	4	0	39	1
ncRNA UTR5	1	0	1	0	0	0	8	0
ncRNA UTR3	0	0	0	0	0	0	6	0
ncRNA splicing	0	0	1	0	0	0	0	0

^1^ex. frmshift del: exonic frameshift deletion

#### Pooled BGI vs Pooled TÜBİTAK

The number of shared and discrepant SNP and indel calls are shown on Table 9. This strategy showed a better correspondence between the two datasets, reducing the contradicting call rate to 0.1%-0.8%. The number of shared and discrepant SNP and indel calls of pooled HaplotypeCaller are also shown on Table 10.

**Table 9:** Comparisons of total and novel SNP and indel intersections of *B*_1_ vs. *T*_1_ and *B*_2_ vs. *T*_2_. *B*_1_, *T*_1_:pooled *S*_1_ calls from BGI and TÜBİTAK datasets; *B*_2_, *T*_2_:pooled *S*_2_ calls from BGI and TÜBİTAK datasets, respectively.

	SNPs		Indels	
	Total	Novel	Total	Novel
*B*_1_ ∩ *T*_1_	3,308,870	41,289	79,948	1,195
*B*_1_ \ *T*_1_	25,857	13,536	651	17
*T*_1_ \ *B*_1_	5,771	483	351	15
*B*_2_ ∩ *T*_2_	3,321,318	51,526	32,391	468
*B*_2_ \ *T*_2_	70,068	46,592	121	11
*T*_2_ \ *B*_2_	1,651	265	231	23

**Table 10:** Comparisons of total and novel SNP and indel intersections of *B*_1_ vs. *T*_1_ and *B*_2_ vs. *T*_2_. *B*_1_, *T*_1_:pooled *S*_1_ calls from BGI and TÜBİTAK datasets using HaplotypeCaller; *B*_2_, *T*_2_:pooled *S*_2_ calls from BGI and TÜBİTAK datasets, respectively.

	SNPs		Indels	
	Total	Novel	Total	Novel
*B*_1_ ∩ *T*_1_	3,551,861	57,010	735,208	49,637
*B*_1_ \ *T*_1_	5,653	1,164	1,396	346
*T*_1_ \ *B*_1_	11,781	1,336	2,743	634
*B*_2_ ∩ *T*_2_	3,595,114	69,416	789,834	55,740
*B*_2_ \ *T*_2_	11,140	1,722	3,687	719
*T*_2_ \ *B*_2_	7,409	961	2,688	597

## 3 Discussion and Conclusion

With the improvements in cost efficiency, speed, and analysis algorithms, HTS platforms are now being considered to be used routinely as part of health care. This assumption prompted a pilot project called ClinSeq [12] that aims to investigate the strength and potential pitfalls of using HTS data in the clinic. However, the HTS technologies continue to evolve and new platforms are introduced almost every month. This, coupled with changes and updates of algorithms to analyze HTS data raises questions about the maturity and robustness of HTS platforms for accurate discovery and genotyping of genomic variants.

In an effort to answer this question, we analyzed the genomes of two individuals, each sequenced twice using the same technology, albeit at different locations. Since our aim was to investigate the maturity of sequencing platforms in this study, we used the same tools to characterize both single nucleotide and short indel variants. Under the assumption of 100% robustness, one would expect to characterize the same set of variants in both sequencing datasets from the same genomes, however, this is not what we found.

We believe multiple factors contribute to this effect. First, since the library preparation is different, one may expect difference in GC% bias, as clearly seen in Table 1 of the manuscript. This leads to differences in read depth over different regions of the genome, which in turn causes discrepancies in variation calls. The GC% effect can also explain the over-representation of repeats and segmental duplications in terms of SNP discrepancies, as common repeats are high in GC content (41.45% GC within common repeats vs 40.33% GC in unique regions), together with difficulties in mapping to repeats and duplications. Second, although the make and model of the sequencing instruments are the same, they are individually different machines, which may account for slight differences in base calling errors. Third, mapping biases against repeats and duplications incur additional problems in terms of mapping and calling. We note that we used the same mapping and calling tools with the same parameters for all datasets in this study, therefore the tools should not be the reason for discrepancies. Although orthogonal methods are needed for definitive validations, we suggest that when there are more than one data set, one should use all the available data for higher accuracy.

Sequencing machines, alignment and genomic variant discovery and genotyping algorithms change rapidly, and one must be careful when interpreting results. Here we demonstrated potential problems that may arise within HTS-based studies. Discrepancies between call sets generated from the same genomes may be complementary false positives and false negatives in each callset, in addition to common genotyping errors. Luckily, much of the differences were found within non-genic regions and common repeats, which are of less importance for most studies.
